# Geographic Variation in Cancer Incidence among Children and Adolescents in Taiwan (1995–2009)

**DOI:** 10.1371/journal.pone.0133051

**Published:** 2015-07-20

**Authors:** Giun-Yi Hung, Jiun-Lin Horng, Hsiu-Ju Yen, Chih-Ying Lee, Yu-Sheng Lee

**Affiliations:** 1 Division of Pediatric Hematology and Oncology, Taipei Veterans General Hospital, Taipei, Taiwan; 2 Department of Pediatrics, Taipei Veterans General Hospital, Taipei, Taiwan; 3 Department of Pediatrics, National Yang-Ming University School of Medicine, Taipei, Taiwan; 4 Department of Life Science, National Taiwan Normal University, Taipei, Taiwan; 5 Department of Anatomy and Cell Biology, School of Medicine, College of Medicine, Taipei Medical University, Taipei, Taiwan; 6 Institute of Public Health, National Yang-Ming University School of Medicine, Taipei, Taiwan; National Health Research Institutes, TAIWAN

## Abstract

**Background:**

Evidence from our recent study suggested that the overall trend for cancer incidence in children and adolescents has been increasing in Taiwan.

**Methods:**

To analyze geographic variations in this trend, cancer frequencies and incidence rates of disease groups were quantified according to geographic areas among 12,633 patients aged <20 years during 1995–2009 by using the population-based Taiwan Cancer Registry. Three geographic levels were defined, namely county or city, region (Northern, Central, Southern, and Eastern Taiwan), and local administrative area (special municipality, provincial city, county-administered city, township, and aboriginal area).

**Results:**

Of the regions, Northern Taiwan had the highest incidence rate at 139.6 per million person-years, followed by Central (132.8), Southern (131.8), and Eastern (128.4) Taiwan. Significantly higher standardized rate ratios (SRRs) were observed in Northern Taiwan (SRR = 1.06, 95% confidence interval [CI] = 1.02–1.10) and at the township level (SRR = 1.07, 95% CI = 1.03–1.11). Of the cities or counties, New Taipei City yielded the highest SRR (1.08), followed by Taipei City (SRR = 1.07). A comparison of the rates in the four regions and the remainder of Taiwan according to cancer type revealed that only the rate of neuroblastomas in Eastern Taiwan was significantly low. Trend analysis showed that the most significant increase in incidence rate was observed at the township level, with an annual percent change of 1.8% during the 15-year study period.

**Conclusions:**

The high rate of childhood cancer in Northern Taiwan and at the township level deserves further attention. The potential impacts of environmental factors on the upward trend of childhood cancer incidence rate in townships warrant further investigation.

## Introduction

Evidence from our recent study revealed that overall rates of cancer in children and adolescents increased by 1% annually during 1995 and 2009 in Taiwan [1]. Additional specific assessments are required to identify the etiological factors that might be implicated in this population. Numerous risk factors and genetic factors are not clearly understood, but may contribute to the observed increasing trends [2–5]. In Taiwan, a study investigating the association between urbanization and childhood leukemia during 1981–1990 indicated that population mixing was one of the risk factors for childhood leukemia [6], a result that is consistent with reports stating that infection is a basis for childhood leukemia [7, 8]. Moreover, other studies have revealed the association between childhood leukemia development and residential exposure to traffic air pollution or petrochemical air pollution [9–11]. A population-based case-control study on the association between radiofrequency exposure and childhood neoplasm revealed a significantly increased risk for all neoplasms in children with higher-than-median radiofrequency exposure to mobile phone base stations [12]. These studies have focused mainly on the linkages between environmental exposures and childhood leukemia or brain neoplasms. Studies on geographic variations of overall childhood cancer or those involving the standard scheme of International Classification of Childhood Cancer (ICCC) [13] are few. In this study, the population-based Taiwan Cancer Registry (TCR) [14] was used to investigate variations and trends in cancer incidence among children and adolescents according to 3 geographic levels and examine the possible relationships between multiple cancers and geographic patterns.

## Materials and Methods

### Data collection

Incidence data for this study were obtained from the TCR, which is organized and funded by the Health Promotion Administration, Ministry of Health and Welfare, Taiwan. All cancers have been registered in the TCR since 1979. In addition, the Taiwan National Health Insurance, a mandatory universal health insurance program launched in 1995 and covering as much as 99% of the population [15], enables insureds to easily access medical services and prompt treatment. After the Cancer Control Act was enacted in 2003, hospitals with a capacity of ≥ 50 beds that provided outpatient and inpatient cancer care were mandated to submit cancer data to the central cancer registry; this Act enhanced the completeness of registration and case ascertainment and improved the quality of cancer data collection [16, 17]. Regarding the quality of TCR data for the age group of 0–19 years [1], according to the quality indicators defined by the International Agency for Research on Cancer (IARC), the percentage of death certificate-only cases decreased by approximately 10%, from 10.3% in 1995 to 0.6% in 2009 (annual average = 6.4% during 1995 and 1999, 1.1% during 2000 and 2004, and 0.4% during 2005 and 2009). Although another indicator of data validity, the percentage of microscopically verified cases (MV%), varied according to the types of cancers, the annual average MV% was 93.7% during the 15-year study period (92.4% in 1995; 94.3% in 2009; range = 91.5%–95.9%). The MV% varied from 92.1% (central nervous system [CNS] neoplasms) to 99.4% (other epithelial neoplasms) for most main ICCC groups, except for hepatic tumors (67.7%) and unspecified malignant neoplasms (group XII of ICCC, 54.0%). Furthermore, another critical indicator for quality of diagnosis, the proportion of cases coded in ICCC group XII, had an average of 0.9% throughout the study period (annual average = 0.7% during 1995 and 1999, 1.1% during 2000 and 2004, and 1.0% during 2005 and 2009). According to these indicators, the data quality of the TCR has remarkably improved over time.

The incidence rates of all cancers in children and adolescents aged 0–19 years and diagnosed from 1995 to 2009 were analyzed. Diagnoses were categorized into 12 main groups according to the ICCC Version 3 (ICCC-3). Only patients diagnosed with malignant tumors were included in the TCR data. We abbreviated 7 of the 12 major ICCC-3 groups as follows: leukemias (leukemias and myeloproliferative and myelodysplastic diseases), lymphomas (lymphomas and reticuloendothelial neoplasms), CNS neoplasms (CNS and miscellaneous intracranial and intraspinal neoplasms), neuroblastomas (neuroblastoma and ganglioneuroblastoma), soft tissue sarcomas (soft tissue and other extraosseous sarcomas), germ cell neoplasms (germ cell tumors, trophoblastic tumors, and neoplasms of the gonads), and other epithelial neoplasms (other malignant epithelial neoplasms and malignant melanomas). The dataset used in this study included case numbers grouped according to year, sex, age, and ICCC-3 and geographic levels in the TCR, which contains no personal information. This study was approved by the Data Release Review Board of the Health Promotion Administration, Ministry of Health and Welfare, which waived the requirement for informed consent.

### Analyses

Age-standardized incidence rates (ASRs) per million person-years according to sex were presented on the basis of the ICCC-3 in the aforementioned main groups. Incidence represents the number of new cancer cases that occur in a defined population, and the incidence rate is the number of such occurrences in a specified period. Thus,

$$\text{Incidence rate}=\frac{\text{Number of new cases of disease}}{\text{Population at risk}}\text{in a specific period}$$

Rates, standard errors (SEs), 95% confidence intervals (CIs), and standardized rate ratios (SRRs) were calculated according to previously published methods by the IARC [18, 19] by using Microsoft Office Excel 2007 software. An ASR is a weighted average of the age-specific (crude) rates, in which the weights are the proportions of individuals in the corresponding age groups of a standard population. The potential confounding effect of age can be reduced when the ASRs computed using the same standard population are compared. In this study, the ASRs were calculated using a direct method of applying the 2000 World Standard Population in 5-year age groups (0–4, 5–9, 10–14, and 15–19 y) and used to examine geographic variations (2011 Taiwan Cancer Registry Annual Report, Appendix 4, the 2000 World Standard Population) [20]. The relative risks of cancer, SRRs (ratio of ASRs), and 95% CIs were calculated to compare the cancer incidence data, in which the SRRs were considered to differ significantly if the estimated 95% CI did not contain 1. To avoid the effect of comparing highly populated cities or counties, regions, or local administrative areas (e.g., New Taipei City, consists of 16.3% of the total Taiwan population), which is itself affected by their contribution, the rate for each region and local administrative area was compared with the remainder of Taiwan (e.g., New Taipei City vs Taiwan − New Taipei City) [18]. The following formula (Smith, 1987) was used:

$${\left({\text{ASR}}_{1}/{\text{ASR}}_{2}\right)}^{1\pm \left({Ζ}_{\alpha /2}/Χ\right)}\text{.}$$

$$\text{where }Χ=\frac{\left({\text{ASR}}_{1}-{\text{ASR}}_{2}\right)}{\sqrt{\left(\text{SE}{\left({\text{ASR}}_{1}\right)}^{2}+\text{SE}{\left({\text{ASR}}_{2}\right)}^{2}\right)}}$$

$$\text{and }{Ζ}_{\alpha /2}=1.96\left(\text{at the 95}\%\text{ level}\right)$$

$$\text{or }{Ζ}_{\alpha /2}=2.58\left(\text{at the 99}\%\text{ level}\right)$$

The method of comparison revealed whether the direct ASRs and ratios (SRRs) were significantly high at the 1% level (++), significantly high at the 5% level (+), nonsignificantly high or low, significantly low at the 5% level (−), or significantly low at the 1% level (−−). Trends were analyzed using the Joinpoint regression model and permutation tests (Joinpoint Regression Program, Version 4.0.4; Statistical Methodology and Applications Branch, Surveillance Research Program, National Cancer Institute, Bethesda, MD, USA) to identify significant changes [21, 22], in which tests as many as 2 joinpoints were produced to express the annual percent change (APC). This software computes trend data and fits the simplest joinpoint model that the data allow. The grid search described by Lerman (1980) was used to fit the segmented regression function, the *P* value of each permutation test was estimated using Monte–Carlo methods, and the overall asymptotic significance level was maintained using a Bonferroni adjustment [22]. The APC was significant if the 95% CI did not include 0.

### Geographic factors

To present comparable results, geographic factors in this study were defined as city or county (n = 22) [23], region (n = 4), and local administrative area (n = 5), as originally designed in the TCR routine analyses for monitoring geographic variations in cancer incidence rates [20]. The 22 cities or counties of Taiwan were grouped into 4 regions: Northern Taiwan (including the cities of Taipei, New Taipei, Hsinchu, Keelung, and Taoyuan and the counties of Yilan and Miaoli), Central Taiwan (Taichung City and Changhua, Nantou, and Yunlin Counties), Southern Taiwan (Chiayi, Tainan, and Kaohsiung Cities and Chiayi, Pingtung, and Penghu Counties), and Eastern Taiwan (Hualien, Taitung, Kinmen, and Lienchiang Counties). Considering that sparse case numbers and population distributions may result in unstable statistics, Kinmen and Lienchiang Counties (offshore islands) were included in the Eastern Taiwan region for statistical analysis. Five local administrative areas were defined as special municipality, provincial city, county-administered city, township, and aboriginal area. The population setting standards for special municipality, provincial city, and county-administered city were ≥ 1 250 000, ≥ 500 000, and ≥ 150 000 people, respectively. Township and aboriginal area were under the jurisdiction of the county. The detailed extent of aboriginal and the aforementioned geographic areas were the same as those previously defined in the TCR. Other information on Taiwan, including land area and census data, were obtained from the Ministry of the Interior, Taiwan [23–26].

## Results

A total of 12 633 patients aged 0–19 years were diagnosed with childhood cancers from 1995 to 2009, yielding ASRs of 139.6, 132.8, 131.8, and 128.4 per million person-years for Northern, Central, Southern, and Eastern Taiwan, respectively (Table 1). Approximately half (n = 5978, 47.3%) of the patients were residents of Northern Taiwan. By contrast, only 2.7% (n = 345) of the patients resided in Eastern Taiwan, which is relatively sparsely populated. The proportion of patients in Central (n = 2933, 23.2%) and Southern Taiwan (n = 3377, 26.7%) was similar, and approximately half of this proportion compared with that in Northern Taiwan. In this analysis, the proportion of ICCC group XII (other and unspecified malignant neoplasms) ranged from 0.6% to 1.1% for the 4 regions, indicating that 98.9%–99.4% of the cancers among the 4 regions involved a specified cancer diagnosis, suggesting that the quality of cancer diagnosis was homogeneously high throughout the 4 regions.

**Table 1:** Annual cancer incidence rates (per million) among children and adolescents aged 0–19 years by geographic regions and International Classification of Childhood Cancer Version 3 group, Taiwan (1995–2009).

					Regions (n = 4)											
	Northern				Central				Southern				Eastern^$^			
ICCC-3 Group	*n*	Crude Rate	ASR*	(95% CI)	*n*	Crude Rate	ASR*	(95% CI)	*n*	Crude Rate	ASR*	(95% CI)	*n*	Crude Rate	ASR*	(95% CI)
I Leukemias	1661	38.3	40.2	(38.3, 42.2)	803	36.1	37.5	(34.8, 40.1)	968	37.6	39.2	(36.7, 41.7)	88	32.9	33.9	(26.8, 41.0)
II Lymphomas	697	16.1	15.7	(14.6, 16.9)	343	15.4	15.1	(13.5, 16.7)	387	15.0	14.6	(13.2, 16.1)	41	15.3	14.7	(10.2, 19.3)
III CNS neoplasms	701	16.2	16.5	(15.3, 17.8)	318	14.3	14.5	(12.9, 16.0)	383	14.9	15.4	(13.9, 17.0)	46	17.2	17.5	(12.4, 22.6)
IV Neuroblastomas	274	6.3	7.2	(6.4, 8.1)	162	7.3	8.3	(7.0, 9.6)	140	5.4	6.4	(5.3, 7.4)	11	4.1	***4*.*5*** ^−^	(1.8, 7.1)
V Retinoblastoma	116	2.7	3.2	(2.6, 3.7)	55	2.5	2.9	(2.1, 3.7)	51	2.0	2.4	(1.7, 3.0)	6	–	–	–
VI Renal tumors	111	2.6	2.9	(2.3, 3.4)	47	2.1	2.3	(1.7, 3.0)	81	3.1	3.5	(2.7, 4.2)	4	–	–	–
VII Hepatic tumors	205	4.7	4.8	(4.2, 5.5)	105	4.7	4.8	(3.9, 5.8)	101	3.9	4.0	(3.2, 4.8)	9	–	–	–
VIII Malignant bone tumors	339	7.8	7.3	(6.5, 8.1)	157	7.1	6.6	(5.6, 7.7)	191	7.4	6.9	(5.9, 7.8)	18	6.7	6.2	(3.3, 9.1)
IX Soft tissue sarcomas	448	10.3	10.1	(9.1, 11.0)	210	9.4	9.2	(8.0, 10.5)	248	9.6	9.4	(8.2, 10.5)	27	10.1	9.5	(5.9, 13.0)
X Germ cell neoplasms	607	14.0	13.8	(12.7, 14.9)	284	12.8	12.6	(11.2, 14.1)	330	12.8	12.5	(11.2, 13.9)	46	17.2	17.1	(12.1, 22.0)
XI Other epithelial neoplasms	630	14.5	13.4	(12.3, 14.4)	331	14.9	13.5	(12.0, 14.9)	385	14.9	13.3	(11.9, 14.6)	30	11.2	10.2	(6.5, 13.9)
XII Other and unspecified malignant neoplasms	46	1.1	1.0	(0.7, 1.3)	33	1.5	1.5	(1.0, 2.0)	32	1.2	1.2	(0.8, 1.6)	2	–	–	–
Not Classified by ICCC or in situ	143	3.3	3.4	(2.8, 4.0)	85	3.8	3.9	(3.1, 4.8)	80	3.1	3.1	(2.4, 3.7)	17	6.4	6.5	(3.4, 9.7)
**Total**	5978	138.0	***139*.*6*** ^**++**^	(136.1, 143.2)	2933	131.8	132.8	(127.9, 137.6)	3377	131.0	131.8	(127.3, 136.3)	345	129.0	128.4	(114.7, 142.0)

Abbreviations: ASRs, age-standardized incidence rates; CI, confidence interval; ICCC-3, International Classification of Childhood Cancer Version 3.

Data include malignant tumors only.

^$^ Counties of Kinmen and Lienchiang (offshore islands) were included in Eastern region for statistical analysis.

* ASRs are per million person-years and were age adjusted to the 2000 world standard population.

^**++**^ Significantly higher than for the remaining area of Taiwan, standardized rate ratio (SRR) = 1.06 (95% CI, 1.02–1.10), *P*<0.01.

^**−**^ Significantly lower than for the remaining area of Taiwan, SRR = 0.61 (95% CI, 0.38–0.98), *P*<0.05.

^–^ Indicates that there were fewer than 10 cases, and the statistic is not displayed in order to avoid presenting unstable data.

### Incidence rate according to the ICCC

Table 1 presents the frequencies and incidence rates of childhood cancer according to the ICCC in the 4 regions. According to ASRs, the 3 most common cancer types were leukemias, CNS neoplasms, and lymphomas for Northern, Central, and Southern Taiwan. Conversely, leukemias (ASR = 33.9 per million person-years), CNS neoplasms (ASR = 17.5 per million), and germ cell neoplasms (ASR = 17.1 per million) were the most common in Eastern Taiwan. The ASRs for germ cell neoplasms were the highest in Eastern Taiwan, compared with those ranging from 12.5 to 13.8 per million in the 3 other regions. However, a comparison of the ASRs in the four regions and the remaining area of Taiwan according to the ICCC groups revealed that only the rate of neuroblastomas (group IV of the ICCC) in Eastern Taiwan was significantly low (SRR = 0.61, 95% CI = 0.38–0.98, *P* < 0.05). The rate of the 11 other ICCC groups in the 4 regions did not differ significantly from that of the remaining area of Taiwan.

### Incidence rate according to city level

**Fig 1:**
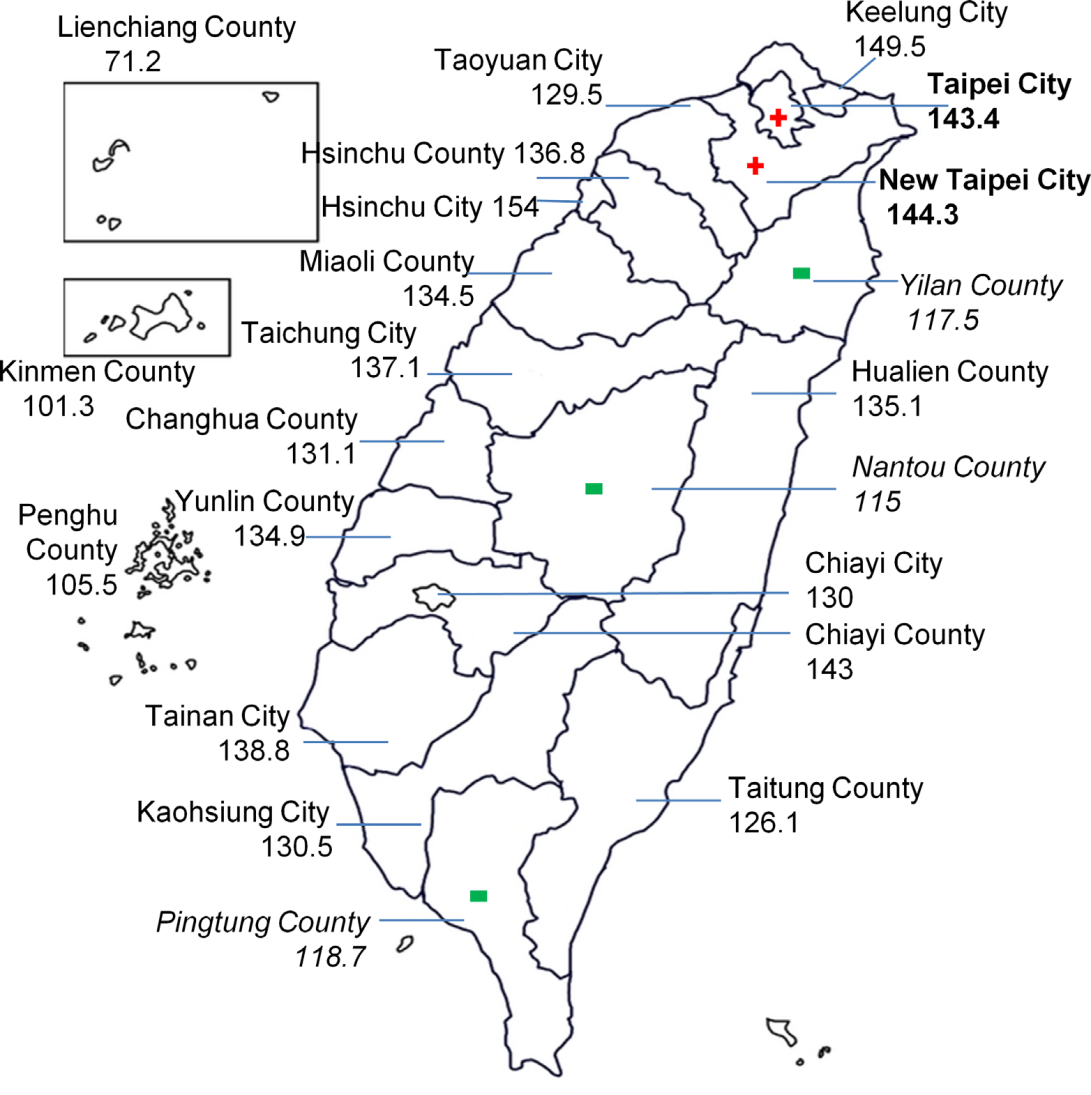
Age-standardized incidence rates (per million person-years) of childhood cancer in patients aged 0–19 years among 22 cities and counties in Taiwan (1995–2009). Reproduced from Taiwan Geographic Name Information Systems [23], the figure is similar but not identical to the original image, and is therefore for illustrative purposes only.

Table 2 and Fig 1 show the case numbers, crude rates, and ASRs of childhood cancer among the 22 cities or counties in Taiwan as well as the averaged population densities and its changes during the 15-year study period of the 22 cities or counties. The 3 highest ASRs were 154.0, 149.5, and 144.3 per million person-years in Hsinchu, Keelung, and New Taipei Cities, respectively. Among the 22 cities or counties, the top 3 yielding the highest incidences of cancer cases were New Taipei City (n = 2179, 17.2%), Taichung City (n = 1532, 12.1%), and Taipei City (n = 1433, 11.3%). By contrast, the regions yielding the lowest relative frequencies were in the offshore islands, including Penghu (0.3%), Kinmen (0.2%), and Lienchiang Counties (0.02%). A comparison of the SRRs of the 22 cities or counties and the remaining area of Taiwan revealed significantly higher rates in Taipei City (SRR = 1.07, 95% CI = 1.01–1.13) and New Taipei City (SRR = 1.08, 95% CI = 1.03–1.13). In addition, the population densities of both cities were among the top 5 in Taiwan (ranked first and fifth among the 22 cities or counties). In contrast to the findings for Taipei City and New Taipei City, significantly lower rates were observed in Pingtung (SRR = 0.87, 95% CI = 0.80–0.95), Yilan (SRR = 0.86, 95% CI = 0.76–0.98), and Nantou Counties (SRR = 0.85, 95% CI = 0.75–0.95), for which the population densities ranked 17^th^, 19^th^, and 20^th^, respectively. No significant difference was determined in the rates of the 17 other cities or counties.

**Table 2:** Incidence rates (per million person-years) and standardized rate ratios (SRRs) of cancer among individuals aged 0–19 years according to cities and counties, Taiwan (1995–2009). Averaged population densities (persons/km^2^) and the changes (%) in population densities of the 22 cities/counties during the 15-year study period were also shown.

City/County (n = 22)	*n*	Crude Rate	ASR^a^	95% CI	SRR^b^	95% CI	Population Density(Persons/km^2^)	Ranking	% Change
***Northern Region***									
Taipei City	1433	142.2	143.4	135.9–150.9	1.07^+^	1.01–1.13	9663.5	1	-1
New Taipei City	2179	142.0	144.3	138.1–150.4	1.08^+^	1.03–1.13	1762.5	5	17.18
Taoyuan City	1053	127.4	129.5	121.6–137.3	0.95	0.89–1.01	1456.7	6	29.8
Keelung City	228	146.8	149.5	129.8–169.2	1.11	0.96–1.27	2910.1	4	5.3
Hsinchu City	256	152.0	154.0	135.1–172.9	1.14	1.00–1.30	3615.2	3	21
Hsinchu County	283	136.3	136.8	120.8–152.7	1.01	0.90–1.14	319.8	15	25
Yilan County	228	117.2	117.5	102.1–132.9	0.86^−^	0.76–0.98	216.2	19	-0.7
Miaoli County	318	133.8	134.5	119.6–149.4	0.99	0.89–1.11	307.8	16	0.3
***Central Region***									
Taichung City	1532	134.5	137.1	130.2–144.0	1.01	0.96–1.07	1121.9	7	16.7
Changhua County	752	131.2	131.1	121.6–140.5	0.97	0.90–1.04	1218.1	8	1.9
Nantou County	254	114.0	115.0	100.8–129.3	0.85^−^	0.75–0.95	131.5	20	-2.9
Yunlin County	395	135.8	134.9	121.5–148.3	1.00	0.90–1.10	573.0	13	-4.1
***Southern Region***									
Chiayi City	150	128.2	130.0	108.9–151.1	0.96	0.82–1.13	4467	2	4.8
Chiayi County	309	144.8	143.0	127.0–159.0	1.06	0.94–1.19	293.9	18	-3.2
Tainan City	1025	137.2	138.8	130.2–147.4	1.03	0.96–1.10	841.5	10	6
Kaohsiung City	1423	129.1	130.5	123.6–137.3	0.96	0.91–1.01	923.6	9	5.8
Pingtung County	434	119.4	118.7	107.5–130.0	0.87^−^	0.80–0.95	325.1	17	-3.2
Penghu County	36	104.4	105.5	70.8–140.2	0.78	0.58–1.04	722.6	12	5.8
***Eastern Region***									
Hualien County	194	136.2	135.1	116.0–154.2	1.00	0.86–1.15	75.8	21	-5
Taitung County	122	125.6	126.1	103.6–148.6	0.93	0.78–1.11	69.2	22	-8.6
***Offshore Islands***									
Kinmen County	27	108.8	101.3	62.6–140.0	0.75	0.54–1.04	418.1	11	97.9
Lienchiang County	2	–	–	–	–	–	291.2	14	69.4
**Total**	**12633**	**134.4**	**135.6**	**133.2–138.0**					

Abbreviations: ASR, Age-standardized incidence rate; CI, confidence interval; SRR, standardized rate (ASR) ratio.

^a^ ASRs were age adjusted to the 2000 world standard population.

^b^ SRR: ASR of the city/county vs. ASR of the remaining area of Taiwan.

^**+**^: Significantly higher than for the remaining area of Taiwan, *P*<0.05.

^**−**^: Significantly lower than for the remaining area of Taiwan, *P*<0.05.

^–^ Indicates that there were fewer than 10 cases, and the statistic is not displayed in order to avoid presenting unstable data.

Census and land data were obtained from the Department of Statistics, Ministry of the Interior, Taiwan [24].

### Incidence rate according to other geographic factors

Table 3 presents the incidence rates and APC of childhood cancer according to geographic factors, including region and local administrative area. Among the 4 regions, only Northern Taiwan exhibited a significantly higher rate (SRR = 1.06, 95% CI = 1.02–1.10, *P* < 0.01) as compared with the remaining area of Taiwan. Regarding the 5 local administrative areas, the highest ASR was observed in townships (141.2 per million), followed by provincial cities (140.8), special municipalities (137.3), county-administered cities (124.5), and aboriginal areas (121.0). Compared with the remaining area of Taiwan, townships (SRR = 1.07, 95% CI = 1.03–1.11) and county-administered cities (SRR = 0.89, 95% CI = 0.86–0.93) yielded significantly higher and lower ASRs, respectively. However, the rates differed nonsignificantly among special municipalities, provincial cities, and aboriginal areas.

**Table 3:** Annual cancer incidence rates (per million) and annual percent changes in cancer incidence rates by geographic factors included region and local administrative area among individuals aged 0–19 years, Taiwan (1995–2009).

Geographic factor	*n*	Crude Rate	ASR^§^	SE	APC (95% CI) 1995–2009	SRR^||^ (95% CI)
**Region (n = 12633)**						
Northern	5978	138.0	139.6^++^	1.82	0.7 (-0.2 to 1.6)	1.06 (1.02–1.10)
Central	2933	131.8	132.8	2.47	1.3 (0.2–2.4)*	0.97 (0.93–1.01)
Southern	3377	131.0	131.8	2.29	1.7 (0.7–2.6)*	0.96 (0.93–1.00)
Eastern	345	129.0	128.4	6.96	-1.2 (-4.4 to 2.0)	0.95 (0.85–1.05)
**Local Administrative Area (n = 12618)**						
Special municipality	2195	135.9	137.3	2.96	1.6 (0.2–3.0)*	1.02 (0.97–1.07)
Provincial city	1653	137.8	140.8	3.50	0.8 (0.0–1.6)	1.05 (0.99–1.10)
County-administered city	3329	122.0	124.5^− −^	2.19	-0.2 (-1.1 to 0.7)	0.89 (0.86–0.93)
Township	5331	141.4	141.2^++^	1.94	1.8 (0.8–2.7)*	1.07 (1.03–1.11)
Aboriginal area	110	124.2	121.0	11.59	-0.1 (-5.0 to 5.2)	0.89 (0.75–1.07)

Abbreviations: APC, annual percent change; ASR, age-standardized incidence rate; CI, confidence interval; SE, standard error; SRR: standardized rate (ASR) ratio.

^§^ ASRs are per million person-years and were age adjusted to the 2000 world standard population.

^||^ SRR: rate vs. rate of the remaining area of Taiwan.

* Indicates statistical significance at the 0.05 level.

^**++**^ Significantly higher than for the remaining area of Taiwan, *P*<0.01.

^− −^ Significantly lower than for the remaining area of Taiwan, *P*<0.01.

### Temporal trends

**Fig 2:**
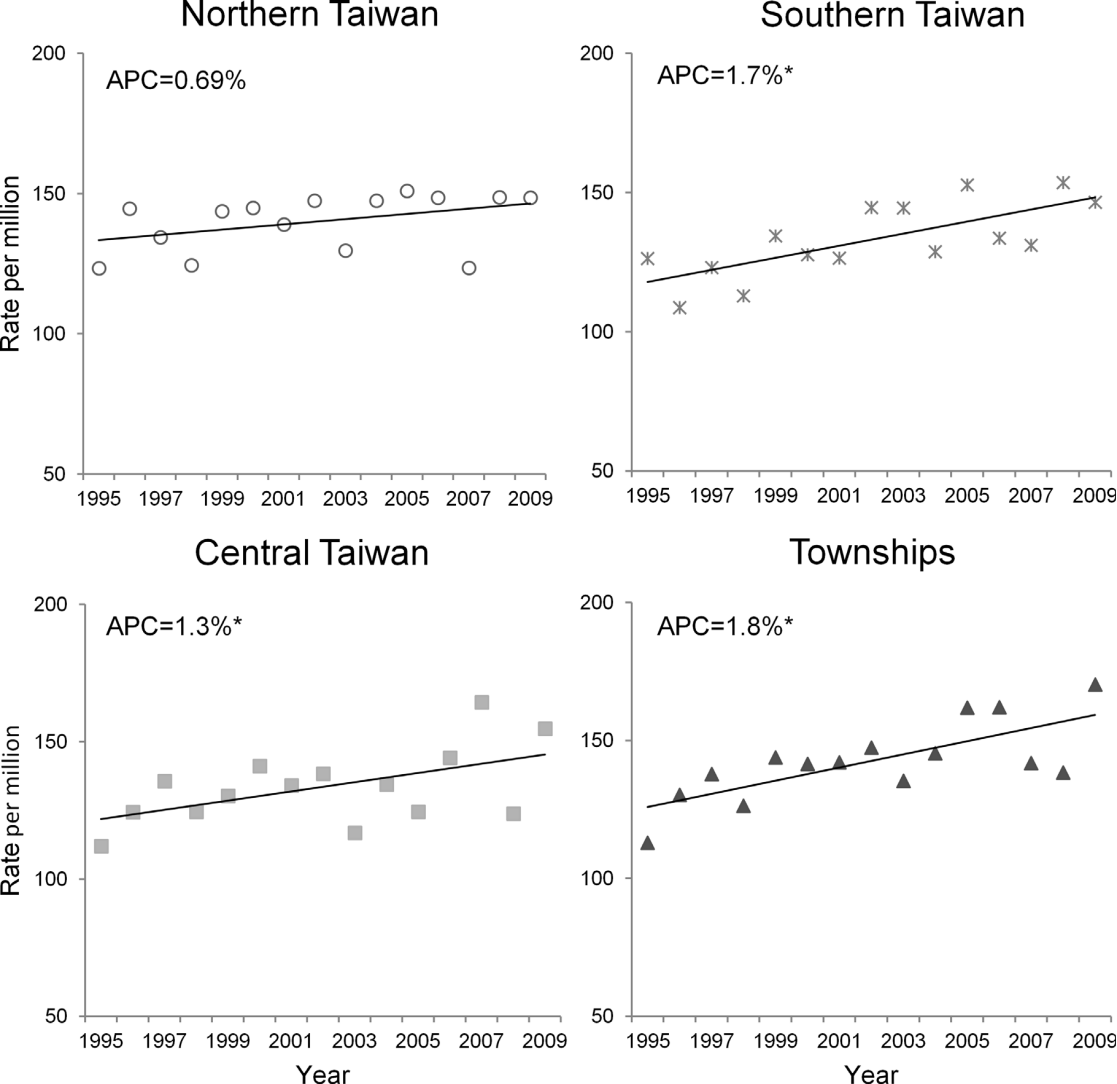
Temporal trends in age-standardized incidence rates of cancer in the patients aged 0–19 years in certain geographic areas (Northern, Central, and Southern Taiwan and in townships) in Taiwan (1995–2009). APC indicates annual percent change. * Statistical significance at the 0.05 level.

Fig 2 and Table 3 show the APCs in ASRs according to geographic factors. During the 15-year study period, the ASRs rose significantly in Southern (APC = 1.7%, 95% CI = 0.7–2.6) and Central Taiwan (APC = 1.3%, 95% CI = 0.2–2.4); however, the changes were nonsignificant in Northern and Eastern Taiwan. Regarding local administrative areas, significantly increasing trends were found in townships (APC = 1.8%, 95% CI = 0.8–2.7), and special municipalities (APC = 1.6%, 95% CI = 0.2–3.0). The variations in ASRs were nonsignificant in provincial cities, county-administered cities and aboriginal areas.

## Discussion

This study is the first to reveal geographic variations in the incidence patterns of childhood cancer according to ICCC-3 groups in Taiwan. Northern Taiwan had significantly higher rates compared with other regions in Taiwan. Further analysis of the city or county level indicated a significantly higher rate in Taipei and New Taipei Cities. In contrast to the significantly higher rates in townships among the local administrative areas, significantly lower rates were observed in county-administered cities. In trend analysis of incidence rates, significant upward trends were observed in Southern and Central Taiwan as well as in townships and special municipalities. The potential factors contributing to the upward trends and higher rates are discussed as follows.

Taiwan has the 10^th^ highest population density in the world (646 persons/km^2^, April 2014), and 98% of the 23 million residents are Han Chinese; 2% are indigenous peoples who originally lived in Eastern Taiwan (Hualien and Taitung Counties) [24]. The findings of significantly higher rates in Taipei City and New Taipei City and the association with population density deserves further attention. Taipei City (i.e., the capital of Taiwan) and adjacent New Taipei City had the first and fifth highest population densities among all cities and counties (9663.5 and 1762.5 persons/km^2^, respectively, Table 2) [25]. Significantly lower cancer rates were observed in Pingtung, Yilan, and Nantou Counties, the population densities of which ranked 17^th^, 19^th^, and 20^th^ (325.1, 216.2, and 131.5 persons/km^2^, respectively). Consistent with previous studies that have indicated that urbanization and a high population density are associated with increased air- and traffic-related pollution and the risk of childhood leukemia in Taiwan [6], our observations may have provided support for the putative association between the population density and the risk of childhood cancer. However, further investigation is warranted to clarify whether the association can be attributed to the high interrelationship among population density, urbanization, lifestyle and socioeconomic status.

In the United States, a childhood leukemia cluster was found in Nevada, and evidence has suggested that infectious etiology is correlated with population mixing [27, 28]. A similar conclusion was deduced from evidence obtained in an investigation of a childhood leukemia cluster in Hong Kong [8]. During the 15-year study period, Taiwan experienced a remarkable increase in population mixing. The number of foreign spouses rose rapidly, at an annual naturalization of 100 times in 2008 (n = 13 230) compared with that in 1995 (n = 129). Of the foreign spouses, 67.62% were from mainland China, Hong Kong, and Macao, whereas 32.38% came from Vietnam, Indonesia, and other countries [26]. The population density of foreign spouses was the highest in Taipei City [25, 26], and New Taipei City had the highest total number of foreign spouses (n = 94 391, April, 2014) among all cities and counties. In addition, the migration of indigenous people was evident during the 15-year study period [24]. The changes in indigenous populations clearly reflected the trend of many indigenous peoples leaving their original living region; the highest increases in indigenous populations were in Northern Taiwan, including Taoyuan County (+31 490 people) and New Taipei City (+24 398 people). The increase in numbers was significantly higher than that in the original living area of indigenous peoples, including Taitung County (+1623 people) and Hualien City (+7556 people). Leukemia accounted for approximately 30% of the total cases and was the most common type of childhood cancer, which increased the possibility of an association between population mixing and the partial contribution of the upward trend of childhood leukemias to the increasing trend of childhood cancer in Taipei and New Taipei Cities.

In early 2014, Taiwan engaged in large-scale antinuclear action. The government was forced to delay the operation of the fourth nuclear power plant because of an upswing in public anxiety arising from the dangers of radiation leakage. In addition, the public showed concerns about the nuclear safety of 2 of the 3 nuclear power plants located in New Taipei City (operating since 1978 and 1981; both are located approximately 30 km from the capital, Taipei City). Although ionizing radiation is one of the known risk factors associated with childhood cancers (acute leukemia, brain tumors, and thyroid carcinoma) [2, 3, 5], evidence supporting the conclusion must show that radiation exposure increases the risk of cancer in children who live near the nuclear facilities compared with control participants. The present analysis revealed a higher rate of childhood cancer in both Taipei and New Taipei Cities; however, Pingtung County, where the third nuclear power plant (operating since 1984) is located, had a significantly lower incidence rate of childhood cancer (SRR = 0.87, 95% CI = 0.80–0.95; Table 2), which does not support the hypothesis that residential proximity to the nuclear power plants leads to an increased risk of cancer. Additional studies are required to measure residential radiation exposure other than background environmental radiation, the dose-response relationship between radiation exposure and risk of childhood cancer, and cancer incidence and mortality rates associated with nuclear facilities in surrounding areas compared with those in other regions in Taiwan.

Townships had higher rates of childhood cancer compared with the remaining area of Taiwan and exhibited the most significant upward trend during the 15-year study period (APC = 1.8%; Table 3). The increasing trends of childhood cancer in Central and Southern Taiwan also warrant further attention. Notably, most of the high-polluting industries, such as the petroleum and petrochemical, iron and steel, and hog and energy industries, are located in the townships or remote areas of Central and Southern Taiwan [29–31]. The long history of environmental pollution can be traced 40 years to when blackfoot disease (arsenic poisoning) was first found in the southwestern coast of Taiwan [32]. Case-control studies involving long-term tracking have reported the association between chronic arseniasis and disease, revealing excessive rates of lung cancer and urinary tract cancer among participants living in arseniasis-hyperendemic areas [33–35]. However, a systemic review including datasets from Taiwan did not support an association between arsenic exposure and childhood cancer [36]. Because of the rarity and diversity of childhood cancer, conducting studies on the relationship with environmental pollution has been considerably difficult and is seldom thorough in Taiwan. Nonetheless, epidemiological studies on the high rate of childhood cancer in townships, particularly in those places subject to possible exposures, are urgently required.

One of the limitations of this analysis is that the statistic power might have been decreased when the cases were grouped into specific cancer types or geographic levels with a low population (e.g., offshore islands, aboriginal areas, and Eastern Taiwan). Confounding factors associated with a retrospective design include changes in disease coding or diagnostic technology. Improvement in the cancer registry over time influences these estimates to some degree. In addition, the socioeconomic levels of the patients, the possible risk factors and comorbidities associated with childhood cancers were not planned or collected in the original setting of the TCR, and thus, etiological studies for evaluating risk factors and complex interactions with covariates for childhood cancers could not be conducted. Childhood cancers accounted for only 1% of the total cancers and were easier to disregard. The inclusion of disease-specific risk factors in the TCR was initiated for some major cancers, but not for childhood cancers.

## Conclusions

The observations in this study reveal the possibility of increased risks of childhood cancers in Northern Taiwan and at the township level, a result that requires further confirmation in other studies. Our results support the implication of association among population density, population mixing, and risk of childhood cancer. Townships had the most evident upward trend of childhood cancer, and the association with environmental pollution deserves further investigation. Based on the high-quality nationwide population-based dataset of the TCR, the analysis of geographic variations in childhood cancer can serve as a basis for answering descriptive questions and provides reliable knowledge of high-risk geographic areas for overall childhood cancer in Taiwan. These preliminary estimates are fundamental but extremely critical for allocating public health resources and additional research plans in Taiwan.
